# Decoupling Junction and Nanosheet Transport in Graphene Networks via Simple DC Temperature‐Dependent Measurements

**DOI:** 10.1002/smll.202509314

**Published:** 2025-11-06

**Authors:** Emmet Coleman, Luke Doolan, Anthony Dawson, Eoin Caffrey, Cian Gabbett, Weimiao Wang, Kevin Synnatschke, Jagdish K. Vij, Jonathan N. Coleman

**Affiliations:** ^1^ School of Physics, CRANN & AMBER Research Centers Trinity College Dublin Dublin Ireland; ^2^ Center for Advancing Electronics Dresden (CfAED) and Faculty of Chemistry and Food Chemistry Technische Universität Dresden 01062 Dresden Germany; ^3^ Department of Electronic & Electrical Engineering Trinity College Dublin Dublin Ireland

**Keywords:** conductivities, inter‐particle, nanosheets, solution‐processed

## Abstract

Printed nanosheet networks are important for various applications in electronics, sensing, and energy storage. Understanding charge transport in such systems requires separately assessing the contributions of both the nanosheets themselves as well as the inter‐nanosheet junctions. However, achieving this using standard electrical characterization methods can be challenging. Here, a broadly applicable method is presented for the separation and extraction of the temperature‐dependent junction resistance (*R*_J_) and nanosheet resistivity (*ρ*_NS_) by combining a simple theoretical model with temperature‐dependent resistivity measurements on networks fabricated using different nanosheet sizes. *R*_J_ is found to be the bottleneck for transport in networks composed of large, thick nanosheets, whereas *ρ*_NS_ begins to dominate in networks composed of smaller, thinner nanosheets. The extracted *ρ*_NS_ shows weak temperature dependence consistent with semiconducting behavior arising from a mixture of Bernal and rhombohedral layer stacking. *R*_J_ exhibits a power–law dependence which is best described by inter‐sheet hopping via delocalized states just above a disorder‐induced mobility edge. This approach enables simultaneous quantification of junction and nanosheet transport in any nanomaterial network. It provides a simple yet powerful method to achieve an extensive understanding of the transport mechanisms, facilitating the design of optimized printed electronic devices.

## Graphical abstract

A simple, broadly applicable method is presented to disentangle junction and nanosheet contributions to charge transport in printed graphene networks using temperature‐dependent resistivity and nanosheet size variation. This approach quantifies both inter‐sheet and intra‐sheet resistance, revealing distinct semiconducting and hopping behaviors, and offers a practical route to optimize nanomaterial‐based electronic devices.

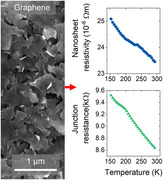

## 1 Introduction

Printed nanomaterial networks, composed of solution‐processed nanostructures such as 0D nanodots/nanoparticles, 1D nanowires/nanotubes, and 2D nanosheets/nanoplatelets, have emerged as a versatile and scalable platform for next‐generation, low‐cost electronic and energy storage devices.^[^ ^1^, ^2^, ^3^, ^4^ ^]^ These networks have found widespread use in areas such as transparent conductors,^[^ ^5^, ^6^ ^]^ flexible electronics,^[^ ^7^, ^8^, ^9^ ^]^ energy storage devices^[^ ^10^, ^11^ ^]^ and sensors,^[^ ^12^, ^13^, ^14^ ^]^ owing to their solution processability,^[^ ^15^, ^16^ ^]^ mechanical flexibility^[^ ^9^, ^17^ ^]^ and tunable electrical properties.^[^ ^15^, ^18^, ^19^ ^]^ Over the last decade, considerable attention has turned to networks of 2D nanosheets. Materials such as MoS_2_,^[^ ^19^, ^20^, ^21^ ^]^ WS_2_^[^ ^9^, ^22^ ^]^ and graphene^[^ ^23^, ^24^ ^]^ have been extensively studied due to their high aspect ratio,^[^ ^19^, ^23^ ^]^ large surface area and tunable bandgaps,^[^ ^25^ ^]^ which enable applications in field‐effect transistors,^[^ ^9^, ^22^ ^]^ photodetectors,^[^ ^9^, ^22^, ^26^ ^]^ printable semiconductors and logic circuits^[^ ^9^, ^19^, ^21^ ^]^ as well as thermoelectric generators.^[^ ^27^, ^28^ ^]^ Among these, graphene nanosheet networks are particularly promising due to the exceptional properties of this prototypical 2D material. The high electrical conductivity of graphene^[^ ^29^ ^]^ enables high‐performance devices, while its outstanding mechanical properties^[^ ^30^ ^]^ enhance the network's mechanical robustness and enables integration into stretchable electronics. Graphene also displays robust chemical stability^[^ ^31^ ^]^ which prevents device degradation over long time periods. These networks have been investigated for use in printable conductors,^[^ ^32^, ^33^, ^34^ ^]^ energy storage,^[^ ^35^ ^]^ EMI shielding,^[^ ^36^, ^37^ ^]^ and gas sensing^[^ ^36^, ^37^, ^38^ ^]^ where maximization or tunability of conductivity is often required.

As with all nanomaterial networks, electrical transport in 2D nanosheet networks is governed by two competing resistive contributions: the resistance of the nanosheets (*R*_NS_, intra‐sheet) and the resistance of the nanosheet‐nanosheet junctions (*R*_J_, inter‐sheet). Intra‐sheet transport typically occurs via bandlike transport in ordered systems or hopping in disordered or defective ones.^[^ ^2^, ^39^, ^40^ ^]^ When charge travels from one nanosheet to the next, it must traverse a nanosheet–nanosheet junction, a process known as inter‐sheet transport. This typically occurs via hopping or tunneling.^[^ ^40^, ^41^ ^]^ Conventional methods for establishing transport mechanisms, such as measuring the electrical conductivity or mobility as a function of temperature, are not ideal for nanomaterial networks as they tend to reflect the rate‐limiting resistive contribution (inter‐ or intra‐sheet) and do not give a complete picture of both inter‐ a intra‐sheet conduction.

Networks of 2D nanosheets (and indeed, all nanomaterials) typically display conductivities or mobilities significantly below those of their constituent materials.^[^ ^40^ ^]^ This tends to be because the inter‐sheet junction resistances are much larger than those of the nanosheets themselves, that is, *R*_J_ >> *R*_NS_.^[^ ^2^, ^40^ ^]^ Under these circumstances, electrical measurements only probe inter‐sheet transport across the junctions, with analysis often assuming the intra‐sheet contribution is negligible.^[^ ^39^ ^]^ However, as we progress toward better networks with larger conductivities and mobilities,^[^ ^21^, ^23^ ^]^ the junction resistances will become comparable to the nanosheet resistance (*R*_J_ ≈ *R*_NS_),^[^ ^21^ ^]^ making it essential to decouple and quantify both inter‐sheet and intra‐sheet charge transport.

Here, we use liquid‐phase exfoliated (LPE) graphene networks as a model system to demonstrate a broadly applicable methodology to measure the temperature‐dependence of both the inter‐sheet junction resistance (*R*_J_) and the intrinsic nanosheet resistivity (*ρ*_NS_). The latter parameter can be combined with the nanosheet thickness to extract the nanosheet resistance (*R*_NS_). We achieve this by performing temperature‐dependent resistivity measurements on a set of networks produced from graphene nanosheets of varying sizes. Combining this data with a simple model allows us to extract the temperature dependence of both *R*_J_ and *ρ*_NS_, providing more complete insight into the transport properties of printed graphene systems. We believe this work represents a significant advancement in methods to facilitate the quantitative understanding of charge transport in nanomaterial networks.

## 2 Results and Discussion

### 2.1 Control and Measurement of Nanosheet Size

Fundamental to this study was the production and isolation of graphene nanosheets with a range of thicknesses. Bulk graphite was exfoliated into graphite/graphene nanosheets by LPE.^[^ ^34^, ^42^ ^]^ This process is known to result in a dispersion of graphene nanosheets with a range of both thicknesses and lateral sizes, but relatively low aspect ratios.^[^ ^42^, ^43^ ^]^ Liquid cascade centrifugation (LCC) is commonly employed to size‐select dispersions of nanosheets by sequentially centrifuging the dispersion at increasing centrifugal speeds/forces.^[^ ^44^ ^]^ After each centrifugation step, larger and heavier nanosheets sediment and are collected. The resulting supernatant contains the smaller unsedimented nanosheets, which are then subjected to the next, higher‐speed centrifugation. This stepwise process produces a series of nanosheet fractions, each enriched with a distinct size range.^[^ ^17^, ^40^, ^45^ ^]^ We note that LCC is widely used, and there is no evidence that it involves selection by any property other than size. Put another way, there is no reason to believe that the size‐selected fractions produced by LCC vary in any meaningful way in terms of basal plane defect density of the nanosheets.

**Figure 1:**
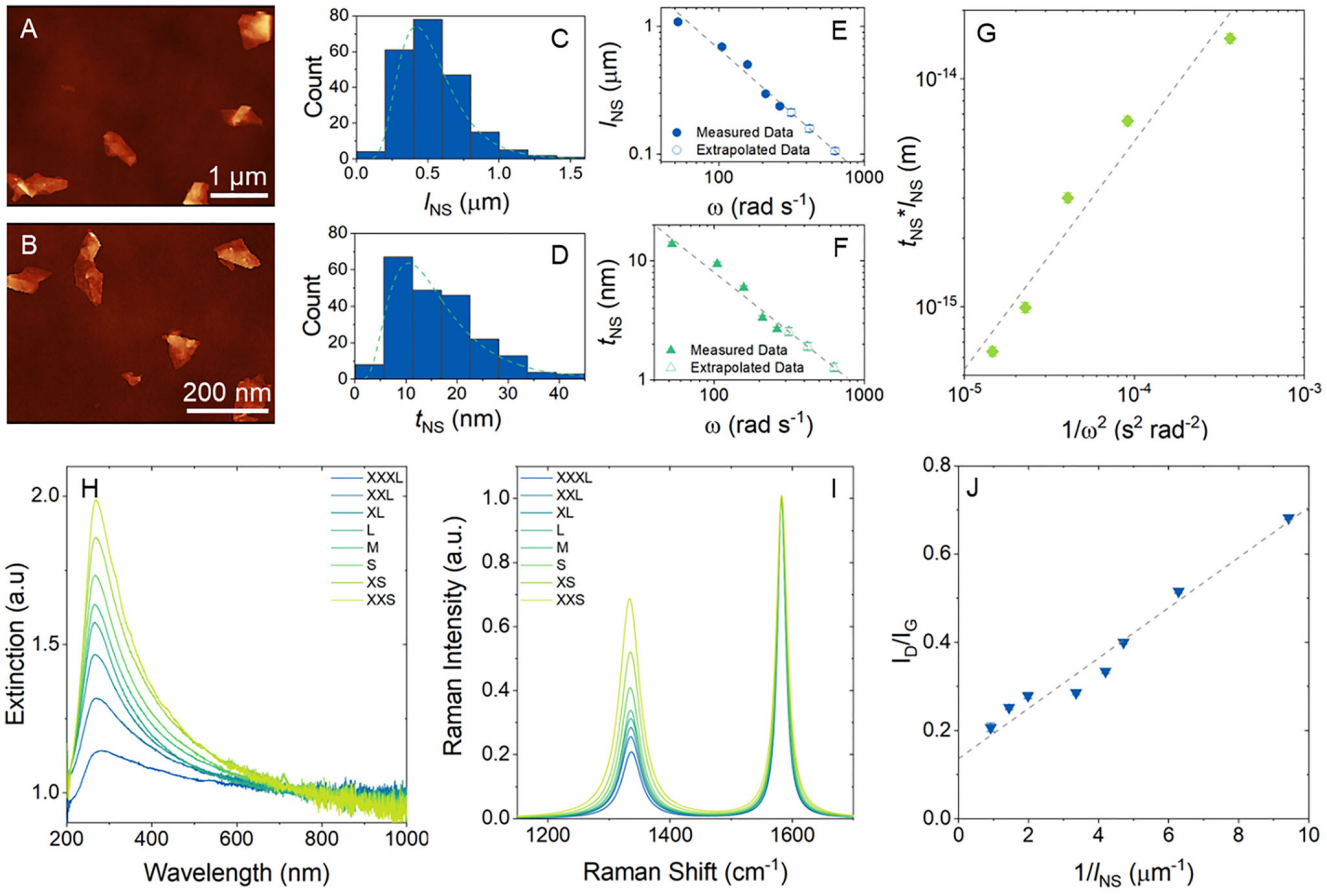
Material Characterization. A,B) AFM images of the largest (A) and smallest (B) fractions. C,D) Example histograms displaying the measured nanosheet lengths (C) and nanosheet thicknesses (D) from AFM statistics for the *t*_NS_ = 5.97 nm sample. E,F) Graphs of the measured and extrapolated nanosheet lengths (E) and thicknesses (F) as a function of centrifugation speed. The lines are a linear fit with a slope −1. G) A plot of ⟨*t*_NS_ *l*_NS_⟩ against ω^−2^ for the measured values showing a linear relationship H) UV/Vis spectra of the various size fractions. I) Raman spectra of the various size fractions showing the characteristic D and G peaks of graphene. J) A plot of *I*_D_/*I*_G_ as a function of 1/*l*_NS_ displaying a linear relationship.

We performed atomic force microscopy (AFM) analysis on a subset of our size‐selected fractions. AFM images, such as those shown in **Figure** **1**A,B, facilitate statistical analysis to measure the distribution of nanosheet lengths (*l*_NS_) and thicknesses (*t*_NS_) in each of the size‐selected fractions (see Figure 1C,D for example histograms and Supporting Information for all statistical data). These histograms yield the mean values of *l*_NS_ and *t*_NS_, which are plotted versus the (upper) centrifugation speed (*ω*) used in the cascade step to generate that fraction (Figure 1E,F). The dispersions subjected to the lower speed centrifugations contain large and thick nanosheets, closer to nanographite than graphene, while dispersions subjected to higher speed centrifugations contain much thinner and laterally smaller nanosheets, consistent with few‐layer graphene.

Recently, Goldie et al.^[^ ^46^ ^]^ applied centrifugation theory to dispersions of nanosheets size‐selected using LCC. They found that the product of mean nanosheet thickness and mean nanosheet length is always inversely proportional to the square of centrifugation speed: *t_NS_l_NS_* ∝ ω^−2^. Figure 1G confirms that our AFM data does indeed follow this trend. Furthermore, ref. [46] shows for systems where the mean nanosheet length scales linearly with the mean nanosheet thickness (as is usually approximately the case for LPE nanosheets),^[^ ^43^ ^]^ both mean nanosheet thickness and mean nanosheet length scale individually with inverse centrifugation speed: *t_NS_* ∝ ω^−1^, *l_NS_* ∝ ω^−1^. This behaviour is confirmed by the fit lines in Figure 1E,F. The confirmation of this trend allowed for the extrapolation and estimation of the nanosheet length (*l*_NS_) and nanosheet thickness (*t*_NS_) for the remaining, smaller fractions (as shown in Figure 1E,F).

UV/Vis spectroscopy of the dispersions displayed the characteristic graphene π–π* transition peak at ≈267 nm as well as the plateau at longer wavelengths,^[^ ^34^, ^47^ ^]^ as shown in Figure 1H. Previous observations have shown that the peak amplitude relative to the plateau increases with decreasing nanosheet thickness.^[^ ^48^ ^]^ This trend was also observed in our UV/Vis spectra, further validating our use of the LCC process.

It is important to ensure that the defect content in the basal plane of the graphene nanosheets does not increase as the nanosheet size decreases. An increased basal plane defect density could reduce the intrinsic conductivity of the nanosheets, thereby affecting the overall conductivity of the network; a key parameter under investigation. To assess this, Raman spectroscopy was performed on the graphene nanosheets (post‐network deposition). Figure 1I shows the Raman spectra of the graphene nanosheets, with the characteristic D (≈1330 cm^−1^) and G (≈1550 cm^−1^) peaks clearly visible.^[^ ^49^, ^50^ ^]^ The G peak is known to arise from the in‐plane stretching of the sp^2^ carbon–carbon bonds, while the D peak originates from a breathing mode of sp^2^ carbon rings and is activated by the presence of defects.^[^ ^51^ ^]^ The intensity ratio *I*_D_ */I*_G_ is commonly used to estimate the level of disorder or defect density within graphene nanosheets. Figure 1J shows that *I*_D_ */I*_G_ increases linearly with 1*/l*_NS_, indicating that the observed defects predominantly originate from the nanosheet edges, rather than within the nanosheet basal plane.^[^ ^48^, ^52^ ^]^ A slope of 56.9 ± 4.1 nm, which is related to the edge‐activated Raman process, and an intercept of 0.137, which is related to the defects present in the parent graphite, are consistent with previous reports.^[^ ^48^ ^]^ We note that the linearity of this graph, and indeed similar graphs previously reported in the literature,^[^ ^48^, ^52^ ^]^ provides strong evidence that the basal plane defect density does not vary from fraction to fraction for liquid phase exfoliated nanosheets size‐selected by LCC. This in turn suggests that basal plane defects do not significantly contribute to variations in network conductivity with nanosheet size.

### 2.2 Network Characterization

**Figure 2:**
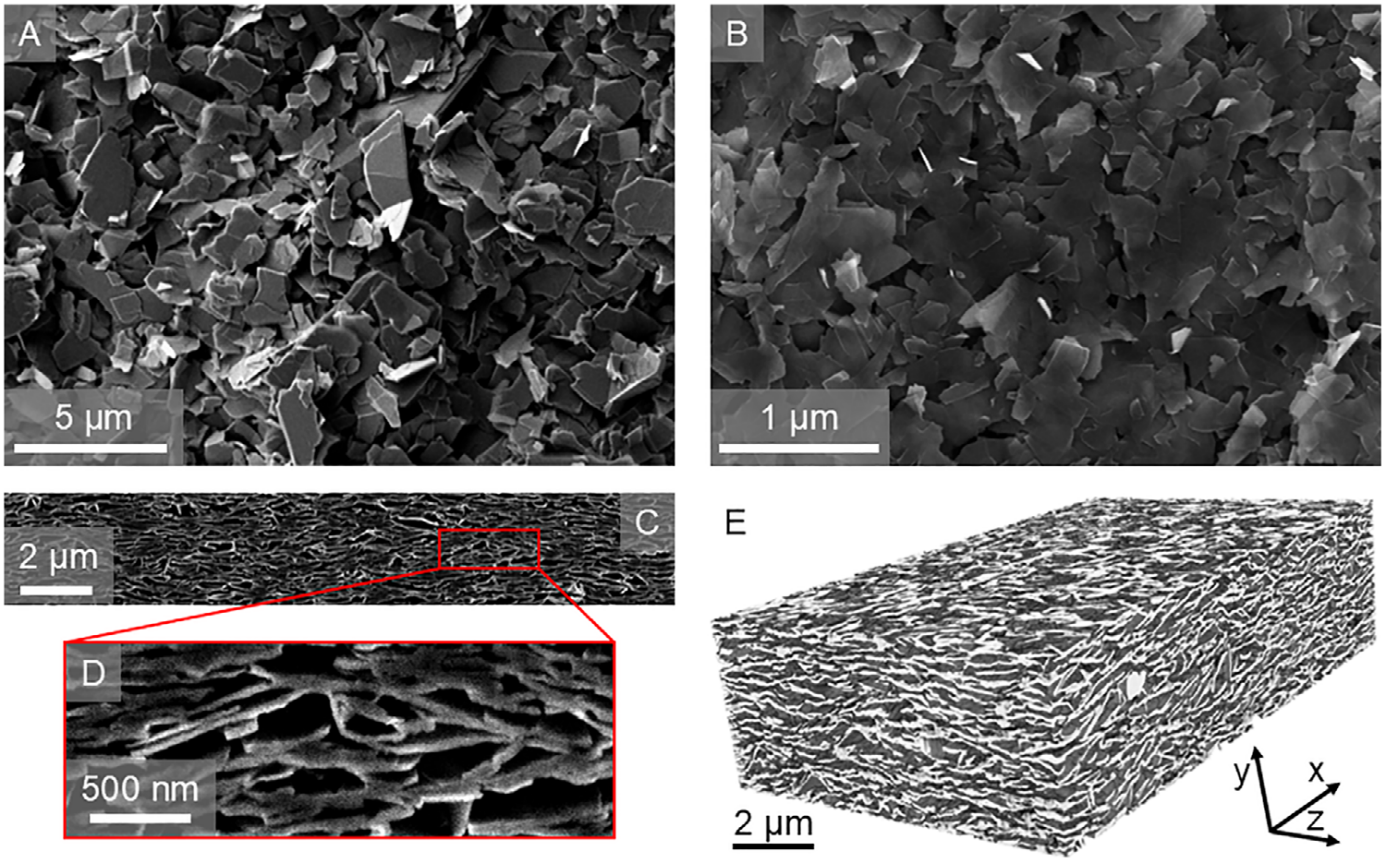
Network Characterization. A,B) SEM images of the top surface of graphene nanosheet networks with nanosheet thicknesses of *t*_NS _= 6 nm (A) and *t*_NS_ = 1.3 nm (B). C) Typical cross‐sectional SEM image of a graphene nanosheet network. D) Close‐up of the network cross‐section showing the disordered nature of the nanosheet‐nanosheet junctions. E) FIB‐SEM tomography image of a section of a network of spray‐coated graphene nanosheets with thicknesses of *t*_NS_ = 3.3 nm.

Networks of graphene nanosheets were produced via spray‐coating.^[^ ^53^ ^]^ All networks were deposited with thicknesses greater than 1 µm to avoid percolative effects (i.e., the thickness‐dependent conductivity observed in LPE graphene networks below ≈100 nm).^[^ ^54^, ^55^ ^]^ **Figure** **2**A,B shows scanning electron micrscopy (SEM) images of the network top surfaces for both larger and smaller nanosheets. In general, spray‐coating resulted in disordered networks composed of randomly oriented nanosheets with point‐like contacts at the nanosheet–nanosheet junctions.^[^ ^45^ ^]^ Figure 2A,B displays this poor conformity at the junctions, particularly for the thicker nanosheets, with minimal overlap between flakes and predominantly point‐like contacts. Figure 2C shows a cross‐sectional image of a graphene network, with the close‐up in Figure 2D again displaying the point‐like nature of the junction areas. Such morphology is consistent with LPE graphene nanosheets,^[^ ^45^ ^]^ and is expected^[^ ^56^ ^]^ to result in high junction resistances, as discussed later.

We note that such point‐like contacts appear to be associated with networks of low aspect ratio nanosheets, such as those produced by LPE and used here. Recent work^[^ ^56^ ^]^ has shown that low aspect ratio nanosheets tend to form jammed systems with point‐like junctions, whereas large aspect ratio nanosheets form aligned networks with large‐area planar junctions. Thus, we believe that point‐like junctions, such as those observed here, will be obtained for networks of LPE nanosheets deposited using almost any printing method.

To characterize the morphology of the entire network, we used nanotomography based on focused ion beam‐scanning electron microscopy (FIB‐SEM), following the method described in ref. [45]. This approach involves sequential milling of the sample with a focused ion beam while imaging the exposed surfaces with SEM, producing a stack of 2D cross‐sectional images. After segmenting these images to distinguish between different materials (i.e., pores and graphene nanosheets), the stack can be reconstructed into a 3D representation of the network, where each voxel corresponds to either a nanosheet or empty space (pore). The typical resolution achieved was 5 nm in‐plane and 15 nm out‐of‐plane, giving a voxel size of 5 × 5 × 15 nm^3^.

Figure 2E shows a representative 3D reconstruction of one of the graphene nanosheet networks (*t*_NS_ = 3.3 nm). In principle, such images should contain all morphological and structural data that is available at the imaging resolution (5 × 5 × 15 nm^3^). This includes details on nanosheet orientation, conduction path tortuosity, pore shape and size, etc. However, for the purposes of this study, we focus on the network porosity (*P*_Net_) which directly relates to the network electrical resistivity (see Equation (2) below). We note that it is somewhat simplistic to reduce all morphological effects to one parameter, *P*_Net_. This approach ignores the wide distribution of pore sizes and the diverse range of flake junctions. Such disorder may lead to second‐order effects such as local variations in current density, meaning the junction resistance is an effective value, representative of a broad distribution of individual junctions. However, we feel the advantages of our simple model, including tractability and alignment with physical intuition, outweigh the issues above.

Using the method described in ref. [45], the mean porosity of the produced networks was determined. Although the network porosity is known to vary slightly with nanosheet size (roughly in the range 40–50%^[^ ^57^ ^]^), to simplify analysis, we approximate the porosity as the mean porosity of all produced networks, which was found to be *P*_Net_ = 45 ± 1%, consistent with previous reports.^[^ ^40^, ^45^ ^]^

### 2.3 Nanosheet Size Dependence

Despite the importance of junctions to the conduction process, the literature contains very little quantitative data on junction resistances. Although conductive AFM^[^ ^58^ ^]^ or the application of micro‐electrodes^[^ ^59^ ^]^ can yield local information on both nanosheet and junction resistances, such methods are not straightforward and cannot be applied to large area printed networks or in‐device measurements. The lack of basic information has hindered printed device development and forced a reliance on trial‐and‐error for device optimization.

To resolve these issues, we recently reported a model that can be used to fit electrical data allowing the extraction of both nanosheet and junction resistance.^[^ ^40^ ^]^ We demonstrated that for a network of 2D nanosheets, the network resistivity *ρ*_Net_ is given by,

(1) $$\rho_{\mathrm{Net}}\;\approx \;\frac{\left[\rho_{\mathrm{NS}}+2t_{\mathrm{NS}}R_{J}\right]}{\left(1\;-\;P_{\mathrm{Net}}\right)}\left[1+\frac{2}{n_{\mathrm{NS}}t_{\mathrm{NS}}l_{\mathrm{NS}}^{2}}\right]$$

where *ρ*_NS_, *t*_NS_, *R*_J_, and *n*_NS_ are the mean nanosheet resistivity, nanosheet thickness, inter‐nanosheet junction resistance, and nanosheet carrier concentration, respectively. Previously, this equation has been shown to predict trends in networks of graphene, WS_2_ nanosheets, WSe_2_ nanosheets, Ag nanosheets, and silver nanowires (AgNWs), and has been used to extract *R*_J_ and *ρ*_NS_ in each of these systems.^[^ ^40^, ^60^ ^]^ In the case of metallic or semimetallic nanosheets, such as graphene nanosheets, where *n*_NS_ is very large, the final term of the equation becomes negligible to give:

(2) $$\rho_{Net}=\frac{\left[\rho_{NS}+2t_{NS}R_{J}\right]}{\left(1\;-\;P_{Net}\right)}$$

This will be the case so long as $n_{\mathrm{NS}}\;>>\;2/\left(t_{\mathrm{NS}}l_{\mathrm{NS}}^{2}\right)$ (that is, for our graphene nanosheets *n*_NS_  > > 2  ×  10^23^ m^−3^), which we expect to always hold since our graphene nanosheets are expected to have a graphite‐like carrier density (≈10^25 ^m^−3^).^[^ ^56^ ^]^

Equation (2) predicts that the network resistivity is directly proportional to the nanosheet thickness. Furthermore, if *ρ*_Net_ is measured as a function of *t*_NS_, the extraction of both *R*_J_ and *ρ*_NS_ will be possible once *P*_Net_ is known. This equation serves as the foundational framework for our analyses, providing a quantitative route to the determination of the role of junctions in LPE graphene networks.

**Figure 3:**
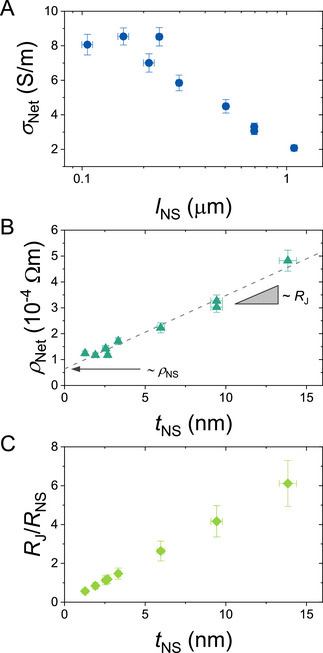
Dependence of Network Electrical Properties on Nanosheet Zize. A) Conductivity of spray‐cast graphene nanosheet networks versus mean nanosheet length. B) Resistivity of spray‐cast graphene nanosheet networks versus mean nanosheet thickness. The line is a fit to Equation (2) which allows for the extraction of *ρ*_NS_ and *R*_J_ from the intercept and slope, respectively. C) Ratio of junction to nanosheet resistance (*R*_J_/*R*_NS_) calculated from values of *R*_J_ and *ρ*_NS_ extracted from the fit in B.

To investigate the effect of nanosheet thickness on the electrical properties of LPE graphene networks, we used the size‐selected graphene nanosheet dispersions produced above to fabricate a set of networks with various nanosheet thicknesses. We then measured the channel length‐dependent resistance of the networks (see Supporting Information for further details) to calculate the contact resistance (*R*_C_) corrected conductivity of the networks. **Figure** **3**A shows this conductivity to fall off with increasing nanosheet length. This behavior, although slightly counter‐intuitive, is a commonly observed phenomenon for networks composed of conducting platelets.^[^ ^17^, ^40^, ^45^, ^61^ ^]^

As predicted by Equation (2), the network resistivity should scale linearly with nanosheet thickness. Figure 3B shows that replotting the data (from Figure 3A) as *ρ*_Net_ versus *t*_NS_ reveals the expected linear behavior. Fitting the data allows us to extract both *ρ*_NS_ and *R*_J_ using the value of *P*_Net_ obtained above. We obtained values of *ρ*_NS_ = (3.5 ± 0.6) × 10^−5^ Ωm, which is broadly consistent with expected values for multilayer graphene or graphite flakes.^[^ ^40^, ^62^ ^]^ In addition, the junction resistance within the networks was determined to be *R*_J_ = 7.8 ± 0.5 kΩ. These values are consistent with the few reports on the junction resistance in LPE graphene networks, which fall in the range 1–100 kΩ.^[^ ^2^, ^40^ ^]^ We note that such values are much smaller than those reported for nanotube networks, which can approach 10 MΩ.^[^ ^63^ ^]^

We can use the known dimensions of the graphene nanosheets, combined with the nanosheet resistivities, to estimate the nanosheet resistance, *R*_NS_, in each size fraction. To do this, we assume the carriers travel, on average, half the lateral length of the nanosheets during intra‐sheet transport,^[^ ^40^ ^]^ leading to *R_NS_*  =  ρ_*NS*_/2*t_NS_*. This allows for the calculation of *R*_J_/*R*_NS_, a parameter which determines if the networks are junction‐limited, nanosheet‐limited, or both, for each of our size fractions. We found values of *R*_J_/*R*_NS_ ranging from 0.56 to 6.11 for the smallest to largest fractions, respectively (Figure 3C). It is important to emphasize that, although *ρ*_NS_ and *R*_J_ are constant over these networks, differences in *t*_NS_ drive differences in *R*_NS_ and, consequently, *R*_J_/*R*_NS_. We note that the networks of smaller, thinner nanosheets have *R*_J_ < *R*_NS_, meaning that they are predominantly limited by the electrical properties of the nanosheets themselves. However, networks of larger, thicker nanosheets are clearly junction‐limited, leaving room for improvement in the future.

From an applications perspective, the ratio *R*_J_/*R*_NS_ can effectively serve as a figure of merit for printed nanomaterial networks. For example, Equation (2) can be rearranged to give: µ_*Net*_  ≈  µ_*NS*_/(1  +  *R_J_*/*R_NS_*). This clearly shows that reducing junction resistance to achieve *R*_J_/*R*_NS_ < 1 yields a network whose mobility approaches that of the nanosheets themselves.^[^ ^21^ ^]^ Comparing our extracted *R*_J_/*R*_NS_ values with those of other relevant systems, such as spray‐coated AgNWs (*R*_J_/*R*_NS_ ≈ 2–30),^[^ ^60^ ^]^ liquid‐interface deposited high aspect ratio MoS_2_ (≈0.2),^[^ ^21^ ^]^ and spray‐coated AgNSs (2–24), graphene (3–14), low aspect ratio WS_2_ (≈100–1000) and MoS_2_ (≈100–1000),^[^ ^40^ ^]^ demonstrates that our spray‐coated LPE graphene networks perform comparably to other spray‐coated conductors but falls behind state‐of‐the‐art liquid interface deposited networks of high aspect ratio materials. This analysis of *R*_J_/*R*_NS_ provides an actionable framework to guide the optimization of future printed electronic devices.

### 2.4 Temperature Dependence of Graphene Network Resistivity

Measuring the temperature‐dependent electrical characteristics of systems such as networks of nanomaterials is crucial to understanding their primary conduction mechanisms. Numerous studies have reported the temperature‐dependent conductivity or resistivity of networks composed of solution‐processed nanomaterials such as graphene, AgNWs,^[^ ^64^ ^]^ SWCNTs^[^ ^39^, ^65^ ^]^ and MoS_2_.^[^ ^41^, ^66^ ^]^ However, these reports investigate the conduction mechanisms of the network as a whole and cannot separate inter‐ from intra‐sheet effects. Consequently, such measurements primarily probe the rate‐limiting step in the conduction process, which is usually inter‐sheet charge transport across junctions. For a broader understanding of the transport within the network, it is necessary to decouple the contribution of the junctions and the nanomaterials themselves. The ability to simultaneously assess the inter‐sheet (junction) and intra‐sheet (nanomaterial) transport would allow a comprehensive understanding of the conduction phenomena within the networks.

**Figure 4:**
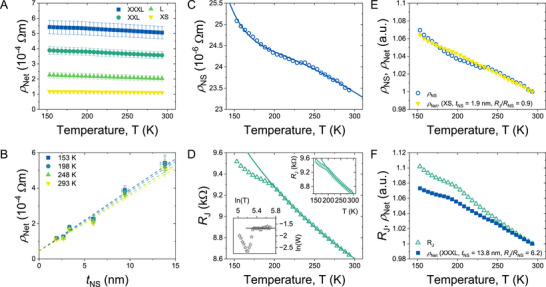
Temperature Dependence of Graphene Network Resistivity. A) Examples of network resistivity versus temperature for various nanosheet thicknesses. B) Examples of network resistivity measured at various temperatures plotted against nanosheet thickness. The lines are fitted to Equation (2). C) The extracted nanosheet resistivity plotted versus temperature. The line is a fit to Equation (6). D) The extracted junction resistance versus temperature for our graphene nanosheet networks. In the top inset, the line is a fit to a 3D‐VRH model, while in the main panel, the line is a power law fit. The bottom inset shows reduced activation energy (W) analysis as described in the text. E) A plot of *ρ*_NS_ and *ρ*_Net_ (both normalized to room temperature values) versus temperature. F) A plot of *R*_J_ and *ρ*_Net_ (both normalized to room temperature values) versus temperature.

We achieve this by measuring the temperature‐dependent resistivity of multiple networks, each with a different mean nanosheet thickness. For technical reasons, we performed two‐probe temperature‐dependent resistivity measurements; we expect the resistivity values to be slightly overestimated, but do not anticipate a substantial effect on the validity of our results. The temperature‐dependent resistivity of a subset of the measured networks is displayed in **Figure** **4**A. The network resistivity of the graphene networks decreases with increasing temperature, as expected for networks of LPE graphene.^[^ ^41^, ^67^ ^]^ In addition, such behavior is observed for many types of graphite above ≈50 K.^[^ ^62^, ^68^ ^]^ It is also worth noting that the resistivity does not vary much within our experimental window, which is typical of both graphite flakes and graphene networks.^[^ ^41^, ^62^, ^68^ ^]^

The temperature dependence of the nanosheet resistivity (*ρ*_NS_) and the junction resistance (*R*_J_) can be extracted from the data using the technique displayed above. Instead of plotting the data in Figure 4A as graphs of *ρ*_Net_ versus temperature for different flake thicknesses, we replot this data as graphs of *ρ*_Net_ versus nanosheet thickness (*t*_NS_), each at a different temperature (Figure 4B). This allows for the calculation of both the nanosheet resistivity and junction resistance at each temperature by fitting each curve using Equation (2), as we did above, enabling the extraction and inspection of the temperature‐dependent *ρ*_NS_ and *R*_J_ individually so that the inter‐ and intra‐nanosheet transport mechanisms can be separately examined.

The extracted nanosheet resistivity as a function of temperature is shown in Figure 4C, while the temperature dependence of the junction resistance is shown in Figure 4D. In both cases the data show weak temperature dependence, with *ρ*_NS_ and *R*_J_ falling slightly as temperature is increased. We note that both curves have subtle but clear differences to their shapes. To test the validity of our method, recall that above we showed networks containing the largest nanosheets are clearly junction‐limited, while networks containing the smallest nanosheets display junction resistances slightly below that of the constituent nanosheets, so are predominantly limited by the nanosheet properties. Therefore, we expect the temperature dependence of *ρ*_Net_ for the largest nanosheets to be similar to that of *R*_J_, and the temperature dependence of *ρ*_Net_ for the smallest nanosheets to be similar to that of *ρ*_NS_. To test this, we plot both *ρ*_NS_ and *ρ*_Net_ for the smallest fraction (*t*_NS _= 1.91 nm), normalized to their room temperature values, versus T in Figure 4E. As expected, both are very similar in shape. In addition, we plot *R*_J_ and *ρ*_Net_ for the largest fraction (*t*_NS_ = 13.9 nm), normalized to their room temperature values, versus T in Figure 4F. Again, both curves are very similar in shape. This strong correlation between the extracted parameters and the corresponding network resistivities provides an independent confirmation of the robustness of our extraction method.

We can now consider both the intra‐sheet and inter‐sheet transport mechanisms, obtained by analysis of the temperature dependence of *ρ*_NS_ and *R*_J_, to determine if they align with expectations. Although many studies on the temperature‐dependent transport within few‐layer graphene/graphite nanosheets exist, there is rarely consensus on the transport mechanism within these systems. However, Zoraghi et al.^[^ ^68^ ^]^ have proposed a model which completely describes the temperature‐dependent resistivity of a range of graphite types and shows it is strongly influenced by the different stacking orders present in the graphite layers. Crucially, the model treats the graphite as semiconducting, in line with multiple previous findings (see ref. [68] and references within, e.g., refs. [69, 70]). They found the temperature dependence of the in‐plane resistance of their graphite samples, and many samples from the literature, can be very well explained over the range 2‐1100 K, assuming three contributions in parallel: two semiconducting phases, attributed to the Bernal and rhombohedral (3R) stacking orders, and a third metallic‐like contribution arising from the interface between the two crystallographic regions. This means the total graphite nanosheet resistivity (*ρ*_t_) can be described by,

(3) $$\rho_{t}{\left(T\right)}^{-1}=\rho_{i}{\left(T\right)}^{-1}+\rho_{s1}{\left(T\right)}^{-1}+\rho_{s2}{\left(T\right)}^{-1}$$

where *ρ*_i_, *ρ*_s1_ and *ρ*_s2_ are the contributions from the interface, 3R and Bernal phase, respectively. It is worth noting that the authors found that the metallic contribution is rarely seen for thinner graphite samples (<30 nm), and when it is, it is only at very low temperatures. Therefore, we can assume its contribution to be negligible in our case. Zoraghi et al. then assumed that each crystalline component (Bernal and 3R) behaves as an intrinsic semiconductor, such that their resistivities are described by,

(4) $$\rho_{s}\left(T\right)=a\left(T\right)\exp\left(\frac{E_{g}}{2k_{B}T}\right)$$

where *E*_g_ is the semiconducting energy gap, *k*_B_ is the Boltzmann constant, and *a(T)* is a mobility‐dependent prefactor which can be written as:

(5) $$a\left(T\right)=\frac{1}{N\left(\mu_{h}+\mu_{e}\right)T^{3/2}}$$

Here, *N* is a temperature‐independent constant and *µ*_h_ and *µ*_e_ are the hole and electron mobilities. Experimental studies^[^ ^71^, ^72^ ^]^ in graphite flakes have shown µ_*h*,*e*_ ∝ *T* ^−3^, leading to *a*(*T*)  =  *a*_0_ *T* ^3/2^. Combining this with Equations (3) and (4) yields the following result:

(6) $$\rho_{t}{\left(T\right)}^{-1}={\left(a_{0,1}T^{3/2}\exp\left(\frac{E_{g,1}}{2k_{b}T}\right)\right)}^{-1}+{\left(a_{0,2}T^{3/2}\exp\left(\frac{E_{g,2}}{2k_{b}T}\right)\right)}^{-1}$$

where the subscripts 1 and 2 refer to the 3R and Bernal components, respectively.

As shown in Figure 4C, Equation (6) fits our data very well across the entire temperature range, confirming the presence of both 3R and Bernal phase stacking in our graphite samples. We note that we could not obtain a good fit considering only one stacking phase. We find the semiconducting bandgaps to be 42 and 195 meV for the Bernal and 3R phases respectively. These values agree well with reported values of 25–61 meV for Bernal and 90–154 meV for 3R phases, respectively.^[^ ^68^ ^]^

We note that it has been shown that Equation (6) applies to graphite down to very low temperatures.^[^ ^68^ ^]^ Thus, it would be interesting to extend the approach described here to temperatures below 10 K in order to confirm whether this equation describes multilayer graphene at low temperature and investigate the interfacial term (*ρ*_i_) which may or may not be present in very thin flakes.

We now turn to the junction resistance which is plotted as a function of temperature in Figure 4D. Unlike the nanosheet resistivity, which reflects the intra‐flake transport, *R*_J_ captures the transport processes across the inter‐nanosheet junctions. To first order, the data above 200 K is consistent with 3D Mott variable range hopping (VRH), as previously observed by Piatti et al.^[^ ^41^ ^]^ for networks of nanosheets very similar to these. 3D‐VRH is consistent with *R* ∝exp (*T*_0_/*T*)^1/4^, where *T*_0_  =  24/(π*k_B_N*(*E_f_*)ξ^3^) and *N*(*E*_f_) is the density of states at the Fermi energy, while *ξ* is the electron localization length.^[^ ^41^ ^]^ Fitting this equation to the *R*_J_ versus *T* data (Figure 4D, top inset) yields a value of *T*_0 _= 75.8 K, which is lower than room temperature and below the temperature range of our experiment. Such behavior has been reported in networks composed of metallic regions when the transport is dominated by inter‐region hopping^[^ ^73^, ^74^, ^75^ ^]^ as is plausible for networks of LPE graphene. In addition, by approximating the density of states via the carrier density, *n*, as *N*(*E_f_*)  ≈  *n*/*k_B_T*, and taking *n* ≈ 10^25^ m^−3^ (refs. [41, 76]), we obtain *ξ* ≈ 14 nm. This value is similar to a previously reported value of *ξ* = 13 nm for an inkjet‐printed LPE graphene network^[^ ^41^ ^]^ and within the range observed for networks of reduced graphene oxide.^[^ ^77^ ^]^

However, identifying the hopping mechanism by directly fitting temperature‐dependent transport data is challenging. For example, different hopping models such as 3D VRH and 2D VRH can regularly give almost equally good fits.^[^ ^78^, ^79^ ^]^ To avoid this problem, the reduced activation energy (*W*) method is often used.^[^ ^41^, ^79^ ^]^ This involves calculating *W*, which is defined here as *W*(*T*)  =   − *d*(ln *R_J_*)/*d*(ln *T*). Plotting ln*W* versus ln*T* should result in a straight line, with a slope that allows unambiguous identification of the appropriate hopping model.^[^ ^77^ ^]^ Such a plot is shown in Figure 4D, lower inset. The high‐temperature portion of this graph is clearly a flat line (slope = 0), which is the hallmark of a power–law relationship: *R_J_* ∝ *T* ^−α^.^[^ ^77^ ^]^ The dip below ln*T *= 5.35 is most probably a transition region. Future work will investigate the lower‐temperature regime with the aim of identifying the dominant inter‐nanosheet transport mechanism for *T* > 200 K.

A power–law relationship has been fitted to the data in the main panel of Figure 4D, yielding a good fit consistent with *α* = 0.1866 ± 0.0005. Such behavior has been observed in networks of reduced graphene oxide,^[^ ^77^ ^]^ nanocrystalline graphene,^[^ ^80^ ^]^ and graphene nanoribbons^[^ ^81^ ^]^ as well as various polymeric systems.^[^ ^82^, ^83^, ^84^ ^]^ Although such power–law behavior can have multiple origins,^[^ ^83^ ^]^ two stand out. Richter et al.^[^ ^81^ ^]^ observed power–law scaling of resistance with temperature in graphene nanoribbon networks. The data was very well described by the so‐called nuclear tunneling mechanism, where the inter‐ribbon hopping was modeled via phonon‐mediated tunneling between biased potential wells separated by a potential barrier. Conceptually, this model appears well suited to inter‐nanosheet transport; however, when applied to nanoribbon networks^[^ ^81^ ^]^ it yields an exponent of *α *≈ 3 and for polymeric systems^[^ ^82^ ^]^ it yields values of *α* in the range 1–7, which are much higher than our value.

The second notable power–law mechanism tends to occur in partially disordered systems in which a mobility edge^[^ ^85^ ^]^ separates extended (delocalized) states at higher energy from localized states at lower energy.^[^ ^84^ ^]^ In such a scenario, a metal–insulator transition (MIT) can occur if the Fermi energy falls below the mobility edge. Power–law scaling has been observed in such systems just above the MIT with expected values of *α* in the range 0.33–1,^[^ ^84^ ^]^ much closer to our value. Kovtun et al.^[^ ^77^ ^]^ studied temperature‐dependent conduction in networks of reduced graphene oxide with different degrees of reduction, observing power–law behavior with exponents varying from 0.19 to 4. Interestingly, the lowest exponents, closest to ours, were found for the most highly graphitized, lowest resistivity networks, similar to those used here. Taken together, these results imply that our nanosheets are not perfectly ordered and display a mobility edge slightly below the Fermi energy. Inter‐sheet hopping occurs via delocalized states just above the mobility edge, which in turn implies the presence of localized states that must be associated with disorder, perhaps basal plane defects or the effects of nanosheet edges. It would be interesting to determine how the mobility edge relates to the band edges associated with the bandgaps of the Bernal and 3R stacked phases discussed above.

## 3 Conclusion

We have presented a method to separate junction resistance from nanosheet resistivity in graphene nanosheet networks. We achieved this by leveraging nanosheet thickness control via LCC and temperature‐dependent resistivity measurements. The linear relationship between network resistivity and nanosheet thickness enabled the extraction of *R*_J_ and *ρ*_NS_, revealing *R*_J_ is the major bottleneck for conduction in networks of thicker nanosheets.

The temperature dependence of *ρ*_NS_ aligns with a two‐phase semiconducting model, with extracted bandgaps consistent with Bernal and rhombohedral stacking in graphite. The junction resistance displays a power–law behavior with *T*, implying hopping to occur via extended states located just above a set of defect‐induced localized states. Our work emphasizes the importance of understanding the charge transport in both the junctions and the nanomaterials in nanonets. This approach can be extended to other 2D material networks, particularly networks of TMDs produced by liquid phase exfoliation which are known to follow Equation (1).^[^ ^40^ ^]^ It should also be applicable to non‐2D nanomaterials such as carbon nanotubes, although a slightly modified model would be required.^[^ ^60^ ^]^ However, for such materials with very low intrinsic resistivity, it may be challenging to extract *ρ*_NT_ due to the very small expected intercept in the graph equivalent to Figure 3B.

In the context of printed electronics, it is of great practical importance to be able to measure both the junction resistance and the nanosheet resistivity. Once these are known, it is immediately possible to identify which factor (junctions or nanosheets) limits conduction. Without this information, it is extremely difficult to improve the conductivity and mobility of networks. In addition, quantifying these parameters allows one to gauge how significant an improvement in conductivity or mobility might be obtained from junction engineering. In addition, we see no reason why this approach could not be used as an in situ operando technique to probe transport in an actual device under working device conditions. We believe this approach to be broadly applicable and will help improve the design of high‐performance printed electronic devices.

## 4 Experimental Section

### Ink Preparation

Nanosheet inks were prepared using LPE.^[^ ^42^ ^]^ Graphite powder (Asbury, Grade 3763) was first sonicated in 80 mL of deionized (DI) water (18.2 MΩ cm) at a concentration of 35 mg mL^−1^ for 1 h. A horn probe sonic tip (Sonics Vibra‐cell VCX‐750 ultrasonic processor) at 55% amplitude, with a pulse rate of 6 s on and 2 s off was used. The resulting dispersion was centrifuged for 1 h at 2684 × g (Hettich Mikro 220 R) to remove potential contaminants from the starting powder. The supernatant was decanted, and the sediment redispersed in 80 mL of DI water and sodium cholate (SC, Sigma Aldrich, >99%) at a concentration of 2 mg mL^−1^. This was sonicated for 8 h at an amplitude of 55% with a 4 s on 4 s off pulse rate. The resulting dispersion was separated into inks of different nanosheet sizes using liquid cascade centrifugation.^[^ ^44^ ^]^ An initial centrifugation step at 28 × g for 2 h was used to isolate unexfoliated material in the sediment. The supernatant was then subjected to additional centrifugation steps at 112 × g, 252 × g, 447 × g, 699 × g, 1006 × g, 1789 × g, and 4025 × g, retaining the sediment at each interval to isolate nanosheet fractions of different sizes. In each case, the sediment was redispersed in a 2 mg mL^−1^ DI: SC solution. The redispersed 112 × g ink was subjected to a further centrifugation step at 28 × g for 1 h to separate it into two size fractions. Each ink was then transferred to isopropanol (IPA, Sigma Aldrich, HPLC grade, 99.9%) for spray coating. To remove the sodium cholate, each dispersion was centrifuged for 2 h at 4052 × g. The supernatant was discarded, and the sediment redispersed in IPA. This step was repeated twice.

### Nanosheet and Ink Characterization

UV–vis optical spectroscopy (Perkin Elmer 1050 spectrophotometer) was used to determine the concentration of the graphene inks using previously reported spectroscopic metrics.^[^ ^48^ ^]^ Each ink was diluted to a suitable optical density, and extinction spectra were recorded in 1 nm increments using a 4 mm quartz cuvette.

Atomic force microscopy was used to measure the nanosheet thickness and lateral dimensions in the size‐selected graphene. A Bruker Multimode 8 scanning probe microscope was used in ScanAsyst mode (non‐contact) in air under ambient conditions using aluminum‐coated silicon cantilevers (OLTESPA‐R3). Substrates for AFM were coated with (3‐Aminopropyl)triethoxysilane (APTES) by soaking them in a solution of APTES and deionized water for 15 min. Substrates were then removed and washed by rinsing with deionized water and pressurized nitrogen repeatedly. Inks as deposited for films were deposited on APTES‐coated SiO2 wafers (5 × 5 mm^2^). A single drop of the dispersion (10 µL) was dropped on the prepared substrate, held for 30 seconds, and then blown off with pressurized nitrogen. The sample was then rinsed with water, followed by isopropanol which was then blown off with pressurized nitrogen. This prevented solvent airdrying on the substrate, creating the coffee ring effect. This process was then repeated with further drops, dense coverage of nanosheets across the sample could be seen under an optical microscope. AFM scan sizes of between 10 × 10 and 20 × 20 µm^2^ were used for shorter and longer nanosheet samples, respectively. Scan rates between 0.35 and 0.7 Hz were used taking either 512 or 1024 lines per image for smaller or larger images, respectively. Using the previous step height analysis, the nanosheet's apparent thickness was converted to layer number and then real thickness.

### Network Deposition

Graphene inks were spray‐coated using a Harder and Steenbeck Infinity airbrush attached to a computer‐controlled Janome JR2300N mobile gantry. The inks were spray coated at a concentration of 0.02 mg mL^−1^ onto ultrasonically cleaned glass slides with pre‐patterned gold electrodes (Temescal FC2000 metal evaporation system) to facilitate electrical measurements. A N2 back pressure of 45 psi, nozzle diameter of 0.4 mm, and stand‐off distance of 100 mm between the nozzle and substrate were used.^[^ ^53^ ^]^ All traces were patterned using stainless‐steel masks while the substrate was heated to 80 °C using a hotplate.

### Network Characterization

Scanning electron microscopy (SEM) of the deposited nanosheet networks was performed using a Carl ZEISS Ultra Plus SEM. Samples were mounted on aluminum SEM stubs using conductive carbon tabs (Ted Pella) and grounded using conductive silver paint (PELCO, Ted Pella). All images were captured at an accelerating voltage of 5 kV using a working distance of 5 mm and a 30 µm aperture. Both the Inlens and SE2 detectors were used for imaging.

An optical profilometer (Profilm3D, Filmetrics) operating in white‐light interferometry mode with a 50× objective lens was used for non‐contact thickness measurements.

Room temperature electrical measurements were performed in ambient conditions using a Keithley 2612A source meter connected to a probe station. Two‐terminal measurements were used to measure the resistivity of the printed LPE graphene networks in the transmission line configuration.

### Raman Spectroscopy

A Renishaw Qontor Raman system, equipped with a 633 nm He/Ne laser was used to evaluate the defect density of graphene nanosheets collected at different rotate speeds. The spectra at 20 different spots were collected for each type of sample, and the data were fitted and averaged with a Lorentzian function.

### Temperature Dependent Conductivity Measurement

The low‐frequency real component of the sample impedance was measured from a frequency of 10 to 1000 Hz in the temperature range 20–120 °C using a broadband α High‐Resolution impedance Analyzer (Novocontrol GmbH, Germany), which utilized a capacitance bridge technique to calculate impedance. The low‐frequency real impedance was expected to be identical to the DC resistance in samples such as these. The samples were placed inside a sample holder which has a fitted Pt 100 Ω resistance temperature sensor in contact with the electrodes. The temperature of the sample was controlled inside a double‐wall cryostat and maintained by a heated N2 jet produced by evaporating liquid nitrogen inside a 50 L dewar (Apollo 50 by Messer Griesheim GmbH). The Quatro temperature controller controlled the power supplied to the dewar and gas heater. The AC measuring voltage applied to the sample was set at 0.5 V.

## Conflict of Interest

The authors declare no conflict of interest.

## Supporting information



Supporting Information

## Data Availability

The data that support the findings of this study are available from the corresponding author upon reasonable request.
