# Supplementary material for: Decoupling Junction and Nanosheet Transport in Graphene Networks via Simple DC Temperature‐Dependent Measurements
Source: Small. 2025 Nov 6;21(50):e09314. doi: 10.1002/smll.202509314 (PMC12710209; doi:10.1002/smll.202509314)
Supplement: Supplementary file 1 — Supporting Information [file SMLL-21-e09314-s001.docx]

Supporting Information for:

Decoupling Junction and Nanosheet Transport in Graphene Networks via simple DC Temperature-Dependent Measurements

Emmet Coleman,^1^ Luke Doolan,^1^ Anthony Dawson,^1^ Eoin Caffrey,^1^ Cian Gabbett^1^, Weimiao Wang^1^, Kevin Synatschke^2^, Jagdish K. Vij^3^ and Jonathan N. Coleman^1^*

*^1^School of Physics, CRANN & AMBER Research Centres, Trinity College Dublin, Dublin 2,*

*Ireland*

*^2^Center for Advancing Electronics Dresden (CfAED) and Faculty of Chemistry and Food Chemistry, Technische Universität Dresden, 01062 Dresden, Germany*

*^3^Department of Electronic & Electrical Engineering, Trinity College Dublin, Dublin 2, Ireland*

*colemaj@tcd.ie (Jonathan N. Coleman); Tel: +353 (0) 1 8963859.

Contents

[S1: Graphene Nanosheet Size Analysis 3](#_Toc204854979)

[S2: Contact Resistance 4](#_Toc204854980)

# S1: Graphene Nanosheet Size Analysis

Supplementary Figure 1.1: **Nanosheet Length Statistics. A-E)** Distributions of nanosheet length, *l*_NS_, measured using AFM for size-selected graphene inks. The distributions are sorted by decreasing *l*_NS_.

Supplementary Figure 1.2: **Nanosheet thickness Statistics. A-E)** Distributions of nanosheet length, *t*_NS_, measured using AFM for size-selected graphene inks. The distributions are sorted by decreasing *t*_NS_.

# S2: Contact Resistance

Supplementary Figure 2.1: **Contact Resistance.** Graph of network resistance, *R*_Net_, versus channel length, *L*_Ch_. The lines are linear fits with the contact resistance extract from the intercept in each case.
